# Supplementary material for: Physical and mental health well-being of COVID-19 recovered patients: A phenomenological study
Source: PLoS One. 2025 Aug 26;20(8):e0324433. doi: 10.1371/journal.pone.0324433 (PMC12380359; doi:10.1371/journal.pone.0324433)
Supplement: Attachment — Submitted filename: grant phemnology two modified_sc.docx [file pone.0324433.s001.docx]

COVID-19 recovered patients' physical and mental health well-being: Phenomenological Study

**Sawsan Abuhammad^1*^, Karem H Alzoubi^2,3^, Omar F Khabour^4^, Shaher Hamaideh^5^, Basheer Khasawneh^5,^ Maryam El-zubi**

^1^Department of Maternal and Child Health, Jordan University of Science and Technology, Irbid 22110, Jordan

^2^Department of Pharmacy Practice and Pharmacotherapeutics, University of Sharjah, Sharjah, UAE.

^3^Department of Clinical Pharmacy, Jordan University of Science and Technology, Irbid, Jordan.

^4^Dept. of Medical Laboratory Sciences, Jordan University of Science and Technology, Irbid 22110, Jordan

^5^Department of Community and Mental Health Nursing, Faculty of Nursing, The Hashemite University, Zarqa, 13133, Jordan

^6^Department of Clinical Pharmacy, Jordan University of Science and Technology, Irbid, Jordan.

**Number of words=** 3400

COVID-19 Recovered Patients' Physical and Mental Health Well-Being: Phenomenological Study

**Abstract**

**Aim:** This study aims to describe the experience of COVID-19 recovered patients' physical and mental health well-being.

**Method:** A qualitative approach was adopted using the unstructured interview protocol. Data were collected from 30 COVID-19 patients who had recovered from moderate to severe form of the disease (hospitalized with oxygen support). Grand tour questions were used during the interviews to fully describe this phenomenon among COVID-19 recovered patients.

**Results:** Four themes emerged from the interviews: physical health (respiratory problems, joint and muscle pain, changes in activity level, and worsening of previous health problems), cognitive health (loss of memory and diminish ability of focusing and changes in cognitive abilities), psychological health (anxiety and nervousness, feeling of loneliness and sadness), and sleeping problems.

**Conclusion :** The experiences that provided by participants in this study validate the presence of Post-COVID-19 in individuals who endured severe cases of COVID-19. The gathered data offer clinicians insights into the subjective, less quantifiable aspects of patients' post-hospitalization experiences.

**Keywords:**Post-COVID-19, Recovered, Mental Health, Physical Health

**Introduction**

The emergence of severe COVID-19 infections has cast a sobering light on the profound and lasting impact that the virus can have on individuals who navigate the treacherous path of severe illness and recovery(1-3). As the global community grapples with the aftermath of the pandemic, a critical imperative arises to closely examine the physical and mental health trajectories of those who have weathered severe bouts of COVID-19(4-6). The study of post-severe infection health is not merely an academic endeavor; it is an urgent call to understand, address, and alleviate the intricate and enduring challenges faced by survivors who have traversed the often arduous journey of severe COVID-19(2, 7).

In addition to the immediate concerns of acute illness, survivors of severe COVID-19 are left grappling with a spectrum of physical and mental health repercussions that demand our attention(8, 9). The consequences extend far beyond the confines of a hospital stay, reaching into the realms of rehabilitation, mental health support, and long-term care(6, 10). By illuminating the intricate dimensions of post-severe infection health, this study aims not only to decipher the complexities of recovery but also to forge a path toward targeted interventions, specialized care, and a deeper comprehension of the factors that influence the trajectory of survivors' well-being(6, 8).

The study of this subject is not only about dissecting the intricacies of physical symptoms or mental health challenges but also about cultivating a nuanced understanding that empowers healthcare providers, policymakers, and communities to provide tailored and empathetic support(11). In studying the physical and mental health of those who have faced severe COVID-19, the authors embark on a journey to enhance the quality of life for survivors, mitigate long-term health risks, and collectively foster a more compassionate and informed response to the aftermath of severe COVID-19 infections. Many survivors of COVID-19 suffer from lingering physical symptoms, often referred to as 'post-covid' symptoms(7, 12). Studying these persistent symptoms helps in identifying and managing specific health issues that may require targeted interventions or ongoing medical attention(13).

The psychological consequences of the pandemic are considerable, and survivors may experience mental health issues (14, 15). Investigating these aspects is critical for implementing appropriate mental health support and interventions to address the emotional well-being of individuals who have faced the physical and emotional strain of COVID-19(16). Moreover, understanding the long-term health consequences of COVID-19 aids in the effective allocation of healthcare resources(17). This knowledge allows healthcare systems to anticipate and meet the evolving needs of survivors, ensuring that medical services are tailored to address the diverse aspects of recovery(18, 19). Public Insights into the post-COVID health landscape contribute to informed public health planning(20) including strategies for vaccination, preventive measures, and health policies that account for the potential long-term health effects on both individual and community levels. This study aims to describe the experience of COVID-19 recovered patients' physical and mental health well-being.

**Method**

In this study, a qualitative approach was adopted using the unstructured interview protocol. Data were collected from 30 COVID-19 recovered patients who experienced moderate to severe form of the disease (hospitalized with oxygen support). Grand tour questions were used during the interviews to fully describe this phenomenon among COVID-19 recovered patients. Unstructured interviews opened the door widely for experience suffering from COVID-19 virus perspectives and allow the researcher to probe to get a complete picture of the issue. Furthermore, qualitative interviews may give some extended statements in which the researcher can make some comparative issues between these statements and the literature review.

**Setting**

The study was conducted among COVID-19 recovered patients at their homes or at an agreed meeting place based on their preferences.

**Data Collections**

The data collections was as the following. 1. Official approval from the hospital directorate. 2. Requesting the list of COVID-19 recovered patients from the hospitals. The list was used to select individuals who satisfied the selection procedure. 3. Conducting one-on-one interviews with prospective respondents to introduce the investigator, explain the study’s objective, and study procedure. There was a discussion of the respondent's right to confidentiality and their withdrawal at their convenience. 4. All required information was explained to the participants. 5. If the individual agrees to participate; they assigned the consent form. Arrangement for time and location for interview was made. (The COVID-19 recovered patients at was interviewed according to the preference. 6. Each interview will last for around 45 minutes to one-hour length 7. Tape-recording and verbatim transcription of the interview was done. Then, translating meaningful quotes into English followed. Back translation and transcribed text comparison were used to achieve translation verification. 8. A follow-up phone call was utilized in exploring new arising issues. The data collection was between 1^st^ of August 2023 and 30^th^ of November 2023

**Data Analysis**

Data was analyzed by content analysis. Data was organized and coded so as to facilitate the identification of recurring patterns.. Themes were searched according to similarities and found the categories. Each transcript of the COVID-19 recovered patient was read thoroughly and analyzed by using Berland’s framework (53). Proposed procedure of Berland’s framework includes seven steps for analyzing data. These stages entail: (1) transcribing, (2) understanding the interview, (3) coding, (4) creating a working analytical approach, (5) using the analytical framework, (6) inserting data into the matrix framework, (7), and lastly data interpretation (54).

Trustworthiness of a qualitative inquiry includes credibility, dependability, confirmability and transferability. In this study, the credibility (validity) of the findings was achieved by two ways. The first way, the researcher will randomly select five of the participants, and contact them with a summary of their experience that they will share. The researcher will ask them if the findings are related to their mentioned information during the interview. If the five participants agree that the findings are their own, this will ensure the researcher that data interpretations are correct. The second way that to be used to validate this study findings was by cross checking with two independent expert doctoral degree researchers, who will independently analyse all the interviews. Then, themes (by the independent analyzers) was compared with the major themes that are to be identified by the original researcher, and an agreement was achieved among all participants.

**Results**

**Demographic Variables**

The following table shows the characteristics of the participants. The participants were consisted of 10 male (33%) and 20 females (66%).

| Table 1: Characteristics of the participants (N=30) | |  |  |
| --- | --- | --- | --- |
| Item | | Frequency | Percent |
| What is your gender? | Female | 20 | 66.7 |
|  | Male | 10 | 33.3 |
| What is your current employment status? | Student | 1 | 3.3 |
|  | Freelance | 1 | 3.3 |
|  | Not work | 14 | 46.7 |
|  | Retired | 8 | 26.7 |
|  | Employee | 6 | 20.0 |
| What is your monthly income in dinars? | 401 to 800 JOD | 13 | 43.3 |
|  | Less than 400 JOD | 15 | 50.0 |
|  | More than 1500 | 2 | 6.7 |
| What is your level of education? | Primary and Secondary | 16 | 53.3 |
|  | Bachelor | 5 | 16.7 |
|  | Diploma | 5 | 16.7 |
|  | Graduate | 3 | 10.0 |
|  | University student | 1 | 3.3 |
| What is your marital status? | Single | 1 | 3.3 |
|  | Married | 29 | 96.7 |
| Where do you live? | Village | 11 | 36.7 |
|  | City | 19 | 63.3 |
| Do you smoke cigarettes or hookah? | No | 28 | 93.3 |
|  | Yes | 2 | 6.7 |
| Have you been infected with Corona more than once? | No | 21 | 70.0 |
|  | Yes 3 times | 5 | 16.7 |
|  | Yes, two times | 4 | 13.3 |
| What kind of vaccine did you receiveه | AstraZeneca | 2 | 6.7 |
|  | Chinese | 12 | 40.0 |
|  | Pfizer | 13 | 43.3 |
|  | I have not received any vaccine | 3 | 10.0 |
| Since when you first got infected with the Corona virus? | 18-24months | 11 | 36.7 |
|  | more than24months | 19 | 63.3 |
| Since when you got infected with the Corona virus the second time (if you were infected more than once)? |  | 17 | 56.7 |
|  | 12-18months | 1 | 3.3 |
|  | 18-24months | 2 | 6.7 |
|  | more than 24months | 9 | 30.0 |
|  | Mild | 1 | 3.3 |
| How would you describe your infection with Corona? | Mild at first time | 3 | 10.0 |
|  | Mild at second time | 6 | 20.0 |
|  | I just affected one time and it was mild | 1 | 3.3 |
|  | I just affected one time and it was severed | 19 | 63.3 |
|  | Equal both times | 1 | 3.3 |
| Which of the following symptoms appeared on you during your infection with Corona [high temperature]] | No | 1 | 3.3 |
|  | Yes | 29 | 96.7 |
| Which of the following symptoms appeared during your infection with Corona [pain in the throat] | No | 2 | 6.7 |
|  | Yes | 28 | 93.3 |
| Which of the following symptoms did you have during your infection with Corona [cough] | No | 6 | 20.0 |
|  | Yes | 24 | 80.0 |
| Which of the following symptoms did you have during your infection with Corona [difficulty breathing]] | No | 1 | 3.3 |
|  | Yes | 29 | 96.7 |
| Which of the following symptoms appeared during your infection with Corona [lack of oxygen]] | No | 5 | 16.7 |
|  | Yes | 25 | 83.3 |
| Have you been admitted to the hospital? | No | 10 | 33.3 |
|  | Yes | 20 | 66.7 |
| Did you need oxygen? | No | 6 | 20.0 |
|  | Yes | 24 | 80.0 |
| Have you been admitted to intensive care? | No | 19 | 63.3 |
|  | Yes | 11 | 36.7 |
| Which of the following health problems persisted for a period of time after you recovered from Corona [feeling short of breath] | No | 5 | 16.7 |
|  | Yes | 25 | 83.3 |
| Which of the following health problems persisted for a period of time after you recovered from Corona [chest pain] | No | 15 | 50.0 |
|  | Yes | 15 | 50.0 |
| Which of the following health problems persisted for a period of time after you recovered from Corona [feeling tired] | No | 2 | 6.7 |
|  | Yes | 28 | 93.3 |
| Which of the following health problems persisted for a period of time after you recovered from Corona [cough] | No | 18 | 60.0 |
|  | Yes | 12 | 40.0 |
| Which of the following health problems persisted for a period of time after you recovered from corona [memory loss]] | No | 7 | 23.3 |
|  | Yes | 23 | 76.7 |
| Which of the following health problems persisted for a period of time after you recovered from corona [delirium]] | No | 26 | 86.7 |
|  | Yes | 4 | 13.3 |
| اWhich of the following health problems persisted for a period of time after you recovered from Corona [feeling depressed] | No | 8 | 26.7 |
|  | Yes | 22 | 73.3 |
| Which of the following health problems persisted for a period of time after you recovered from Corona [feeling anxious] | No | 3 | 10.0 |
|  | Yes | 27 | 90.0 |
| Which of the following health problems persisted for a period of time after you recovered from Corona [repeated diarrhea] | No | 19 | 63.3 |
|  | Yes | 11 | 36.7 |
| اWhich of the following health problems persisted for a period of time after you recovered from Corona [feeling tired]] | No | 3 | 10.0 |
|  | Yes | 27 | 90.0 |
| Which of the following health problems persisted for a period of time after you recovered from corona [sleep disorders]] | No | 1 | 3.3 |
|  | Yes | 29 | 96.7 |
| Which of the following health problems persisted for a period of time after you recovered from corona [joint pain]] | No | 3 | 10.0 |
|  | Yes | 27 | 90.0 |
| Which of the following health problems persisted for a period of time after you recovered from corona [muscle pain] | No | 2 | 6.7 |
|  | Yes | 28 | 93.3 |

**Themes**

The are four themes that emerged from the interviews. These themes are physical health, cognitive health, psychological health, and sleeping problems.

**Physical Health**

The first theme that emerged from the interviews is physical health that include respiratory problems, joint and muscle pain, changes in activity level, and worsening of previous health problems.

**Respiratory problem**

Most of the participants agreed that they suffered from respiratory problems after recovered COVID-19. For example, F (75) “I feel suffocation, shortness of breath, and difficulty sleeping all night because of my breathing”. F (48) “I feel short of breath, but most of all when I sleep”. Another example M (52) “My condition is worse than those who smoke. My chest is ruined by the Corona virus, I could not take a breath”

**Joint and Muscle Pain**

Many of the participants mentioned suffering from joint and muscle pain. For instance, M(63) “ I always complain of muscle and joint pain”. Moreover, M (52) said “I always feel tired in the muscles and joints and pain in the whole body. When you put your hands on the meat, it becomes painful in its place. The whole body feels as if it is weak. My hands and running all hurt. I go and take needles until the pain subsides”. F(53) “I have pain in my joints and muscles. I don’t know if it is from the joints or from the Corona virus. My problem is worsening, no medication could help me to decrease my pain”

**Change in activity level**

Many participants stated changes in activity level. For example, F (49) “when I walk, for example, or when I go up a flight of stairs, or even sometimes when I talk or do any simple effort, I feel tired and I feel tired for almost a whole year like this, and I go back to the hospital because of this”. Additionally, F (48) The feeling of fatigue after it is still present: I mean, before the Corona virus, I was very active, but now, if I do anything quickly, I get tired, and I sit and rest until the fatigue goes away, suddenly I feel tired. Moreover, M (65) “ if I want to go up the stairs, I take one pull and stop, relax and then continue to go, I don’t keep watching”.

**Worsening of the physical problems**

Many people were mentioned worsening of the physical problems. For example, F(75) “my ears became blocked and I stopped listening. I had difficulty hearing the phone. I went to see the doctor and he told me that my ear nerve was tired. It was there before the Corona virus and it has increased”. Moreover, F(41) My body can no longer tolerate any illness. With just a simple flu, he feels tired, not like he used to. At first, he was fine for a day, two days, or three. Now the almonds are expanding”. F(75) There is nothing but fatigue and pain in the legs, muscles, and joints. This pain was present before the Corona virus, but after the Corona virus it increased a lot to the point that I was no longer able to walk on them. On the walker, I walk. F(53) I have discs in the first place, but after the Corona virus it increased. I mean, the joints increased. I have pain in my fingers and I run under my back a lot.

**Cognitive Health**

Another theme that emerged from the interviews are cognitive health that include subheadings such as loss of memory and diminish ability of focusing and changes in cognitive abilities.

**Loss of memory and diminish ability of focusing**

Many participants stated loss of memory and diminish ability of focusing**.** For example, F(58) “I have lost a little memory, and my memorization is slow because I like to memorize the Quraan. Additionally, F(58) “It is different from before, and sometimes I go to get something that I forget again, and sometimes I look for the phone while I am talking about it. M (57) “I forget a lot, and there are things I notice. For example, when you pass in front of me and talk to me after you leave, I forget what you look like. I remember the name, but I forget the appearance. Vitamin B12 and D are normal, but there is memory loss.”

**Change in cognitive abilities**

Some participants agreed that they are suffering of change in their cognitive abilities. For example, M(55) “I used to have a very strong memory, but after Corona I became M (55). I feel like I forgot, and my memory changed by less than half. Moreover, M(58) “I became delirious. Sometimes I used to talk about things they told me that did not exist, but I would get better, thank God”.

**Psychological Health**

Another theme that emerged from the interviews is psychological health and that include anxiety and nervousness, feeling of loneliness and sadness

**Anxiety and Nervousness**

Many participants agreed of suffering of anxiety and nervousness. For example, F(58) “I quickly became nervous. Even my children would tell me, “What’s wrong with you, Mama?” I tell them not to talk to me” Moreover, . F (69) “ The feeling of anxiety that is always with me , especially at night”. F(68) “Anxiety and the impact of the Corona virus on my body are huge, it is also impacted my sleeping in very bad way”. F(61) “I remained nervous. You know life and its circumstances, and youth and circumstances pass by one person. I stopped thinking about anything for a long time. I began to wish that why I had not died like the people who died of the Corona virus”.

**Feeling of Loneliness and Sadness**

Many participants agreed that they are feeling loneliness and sadness. For example, M (73) “ I always feel sad and depressed because I feel I am a stranger to my sons and daughters”. Also, M (52) “I always like to be alone and I love isolation. Sometimes I feel sad and depressed.

**Sleeping Problems**

Almost all participants agreed that they are suffering from sleeping problems. For example, F (69) “ I do not sleep well. I keep thinking about things that are not there. I sleep a little and then someone finds me awake at night. My sleep is intermittent and not continuous. Moreover, M (65) hours in the summer. Now, after I got sick, my sleep is intermittent. I sleep for an hour or an hour and a half. I don’t know how to go back to sleep. I feel anxious. Sometimes I stay up for two or three hours alone to sleep and wake up at night every two hours. F(68) I have trouble sleeping, and I notice that if I wake up at night I don’t know how to sleep again. F(45) I do not sleep at all, even if I sleep, it is not continuous, not deep sleep, and I am very anxious. Sometimes I even think about going to the doctor to write me a sleep aid. I stay on the phone to make myself feel sleepy.

**Discussion**

This qualitative study delved into the recovery journey of individuals diagnosed with COVID-19 and required admission, aiming to enhance our quantitative comprehension of post-discharge results. Our results affirm these individuals enduring physical and psychological symptoms. Through this study, the authors revealed that these individuals in our study encountered a shift in their perceptions of their bodies.

The first theme that emerged from our study is physical health that include respiratory problems, joint and muscle pain, changes in activity level, and worsening of previous health problems. Similarly, deterioration in physical well-being was primarily observed in aspects such as physical role functioning, mobility, and regular activities, which are linked to engagement in work and everyday tasks(21, 22). Furthermore, a decrease in physical capacity was evident, as indicated by post-COVID- 19 results being lower than the typical average of >650 meters in healthy adults(23, 24). In a study, muscle strength was assessed three months after recovering from COVID-19, revealing that 18% of individuals who were in the ICU developed weakness(25). Survivors of COVID-19 have also been observed to experience muscle weakness and acute sarcopenia(26). Paneroni et al. discovered quadriceps weakness in 86% and biceps brachialis weakness in 73% of individuals infected with the coronavirus upon hospital discharge(27). This underscores the significance of assessing muscle strength during follow-up (28, 29).

Many participants in our study mentioned decreased of physical functions. In agreement to our study, studies showed that both patients who were hospitalized (in ward and ICU settings) and those who were not hospitalized exhibited a decline in function and decreased physical capacity during the 1- and 3-month follow-up periods after recovering from COVID-19(30, 31) . Consequently, the diminished physical capacity and function observed in individuals recovering from COVID-19 may also arise from physical inactivity resulting from quarantine, social distancing, and isolation measures.

In our study, many participants mentioned suffering from many mental health issues that include mental symptoms such as anxiety and depression. Similarly, many studies reviewed that reported anxiety symptoms were identified as the predominant mental issue commonly observed in post-COVID-19, with prevalence rates ranging from 7% to 64%(4, 32-34). Reported depression symptoms emerged as the second most prevalent psychological issue, with rates varying between 4% and 31% for periods exceeding one month post-recovery. Upon hospital discharge, both depression and anxiety symptoms were reported by 32% of patients in Italy and 41% in Iran. Another notable post-COVID-19 mental health issue was PTSD, with rates ranging from 13% to 47%. Sleep problems were also prevalent, with rates between 18% and 31%, and cognitive-functional issues were reported in 17% to 4 % of post-COVID-19 individuals, particularly among ICU survivors(11, 35).

In our study, many participants were mentioned impact of disease on their memory. Accordingly in previous studies, memory were moderately decreased in 58% and severely decreased in 19 % of COVID-19 survivors (36). Many participants suffered from mental health issues after recovering from the infection (37). Sleep difficulties, especially insomnia, were identified as a significant mental health challenge following the COVID-19 pandemic, manifesting in both short-term and long-term phases(38). The physiological and psychological stress during the acute phase of the infection were proposed to activate mechanisms leading to the release of pro-inflammatory cytokines, disrupting metabolic and cardiovascular functions associated with sleep(39). Researchers suggested that sleep issues may negatively impact emotional and attentional regulation, resulting in irritability and difficulties concentrating. Additionally, other studies highlighted sleep problems as a primary complication reported by individuals recovering from COVID-19(40, 41).

**Conclusion**

The experiences that provided by participants in this study validate the presence of Post-COVID-19 in individuals who endured moderate to severe cases of COVID-19. The gathered data offer clinicians insights into the subjective, less quantifiable aspects of patients' post-hospitalization experiences. The persistence of symptoms and the sense of isolation set off a self-perpetuating cycle fueled by fear and social stigma. Nonetheless, individuals also initiate recovery processes that enable them to gradually reintegrate into their normal life trajectories. The speed at which these mechanisms are activated universally, and whether rehabilitation can break this detrimental cycle by alleviating lingering symptoms, remains a topic for further exploration.

**References**

1.	Abuhammad S, Alzoubi KH, Khabour O. Fear of COVID‐19 and stigmatization towards infected people among Jordanian people. International Journal of Clinical Practice. 2021;75(4):e13899.

2.	Gharaibeh BA, Abuhammad S, Haneyah O, Mehrass AA-KO. Role of inflammation in determining the severity of COVID-19 infection in patients with diabetes: A comparative study. Medicine. 2023;102(50):e36641.

3.	Post-COVID-19 global health strategies: the need for an interdisciplinary approach. Aging clinical and experimental research. 2020;32(8):1613-20.

4.	Abuhammad S, Khabour O, Alzoubi K, Hamaideh S, Khassawneh B, Alsmadi B, et al. Mental health of COVID-19 recovered individuals: A national study from Jordan. Electron J Gen Med. 2023; 20 (2): em451. 2023.

5.	Abuhammad S, Khabour OF, Alzoubi KH, Hamaideh S, Khassawneh BY, Mehrass AA-KO, et al. Health-Related Quality of Life for Jordanian-Recovered Individuals During Post-COVID-19 Era: A Cross-Sectional Study. Patient preference and adherence. 2023:1303-10.

6.	Delbressine JM, Machado FV, Goërtz YM, Van Herck M, Meys R, Houben-Wilke S, et al. The impact of post-COVID-19 syndrome on self-reported physical activity. International journal of environmental research and public health. 2021;18(11):6017.

7.	Hasenoehrl T, Palma S, Huber DF-X, Kastl S, Steiner M, Jordakieva G, et al. Post-COVID: effects of physical exercise on functional status and work ability in health care personnel. Disability and rehabilitation. 2023;45(18):2872-8.

8.	Figueiredo EAB, Silva WT, Tsopanoglou SP, Vitorino DFdM, Oliveira LFLd, Silva KLS, et al. The health-related quality of life in patients with post-COVID-19 after hospitalization: a systematic review. Revista da Sociedade Brasileira de Medicina Tropical. 2022;55.

9.	Alzueta E, Perrin PB, Yuksel D, Ramos-Usuga D, Kiss O, Iacovides S, et al. An international study of post-COVID sleep health. Sleep Health. 2022.

10.	Cindrich SL, Lansing JE, Brower CS, McDowell CP, Herring MP, Meyer JD. Associations between change in outside time pre-and post-COVID-19 public health restrictions and mental health: brief research report. Frontiers in public health. 2021;9:619129.

11.	Garrigues E, Janvier P, Kherabi Y, Le Bot A, Hamon A, Gouze H, et al. Post-discharge persistent symptoms and health-related quality of life after hospitalization for COVID-19. Journal of Infection. 2020;81(6):e4-e6.

12.	Perrin R, Riste L, Hann M, Walther A, Mukherjee A, Heald A. Into the looking glass: Post-viral syndrome post COVID-19. Medical hypotheses. 2020;144:110055.

13.	Oronsky B, Larson C, Hammond TC, Oronsky A, Kesari S, Lybeck M, et al. A review of persistent post-COVID syndrome (PPCS). Clinical reviews in allergy & immunology. 2023;64(1):66-74.

14.	Pierce JD, Shen Q, Cintron SA, Hiebert JB. Post-COVID-19 syndrome. Nursing research. 2022;71(2):164-74.

15.	Malik P, Patel K, Pinto C, Jaiswal R, Tirupathi R, Pillai S, et al. Post‐acute COVID‐19 syndrome (PCS) and health‐related quality of life (HRQoL)—A systematic review and meta‐analysis. Journal of medical virology. 2022;94(1):253-62.

16.	El Sayed S, Gomaa S, Shokry D, Kabil A, Eissa A. Sleep in post-COVID-19 recovery period and its impact on different domains of quality of life. The Egyptian Journal of Neurology, Psychiatry and Neurosurgery. 2021;57:1-10.

17.	Yong SJ. Long COVID or post-COVID-19 syndrome: putative pathophysiology, risk factors, and treatments. Infectious diseases. 2021;53(10):737-54.

18.	Anaya J-M, Rojas M, Salinas ML, Rodríguez Y, Roa G, Lozano M, et al. Post-COVID syndrome. A case series and comprehensive review. Autoimmunity reviews. 2021;20(11):102947.

19.	Maltezou HC, Pavli A, Tsakris A. Post-COVID syndrome: an insight on its pathogenesis. Vaccines. 2021;9(5):497.

20.	Pavli A, Theodoridou M, Maltezou HC. Post-COVID syndrome: Incidence, clinical spectrum, and challenges for primary healthcare professionals. Archives of medical research. 2021;52(6):575-81.

21.	Daher A, Balfanz P, Cornelissen C, Müller A, Bergs I, Marx N, et al. Follow up of patients with severe coronavirus disease 2019 (COVID-19): Pulmonary and extrapulmonary disease sequelae. Respiratory medicine. 2020;174:106197.

22.	Temperoni C, Grieco S, Pasquini Z, Canovari B, Polenta A, Gnudi U, et al. Clinical characteristics, management and health related quality of life in young to middle age adults with COVID-19. BMC infectious diseases. 2021;21(1):1-10.

23.	Camarri B, Eastwood PR, Cecins NM, Thompson PJ, Jenkins S. Six minute walk distance in healthy subjects aged 55–75 years. Respiratory medicine. 2006;100(4):658-65.

24.	Theofilou P. Quality of life: definition and measurement. Europe's journal of psychology. 2013;9(1).

25.	Van Aerde N, Van den Berghe G, Wilmer A, Gosselink R, Hermans G. Intensive care unit acquired muscle weakness in COVID-19 patients. Intensive care medicine. 2020;46:2083-5.

26.	Disser NP, De Micheli AJ, Schonk MM, Konnaris MA, Piacentini AN, Edon DL, et al. Musculoskeletal consequences of COVID-19. The Journal of bone and joint surgery American volume. 2020;102(14):1197.

27.	Paneroni M, Simonelli C, Saleri M, Bertacchini L, Venturelli M, Troosters T, et al. Muscle strength and physical performance in patients without previous disabilities recovering from COVID-19 pneumonia. American journal of physical medicine & rehabilitation. 2021;100(2):105-9.

28.	Jolley SE, Bunnell AE, Hough CL. ICU-acquired weakness. Chest. 2016;150(5):1129-40.

29.	Schweickert WD, Hall J. ICU-acquired weakness. Chest. 2007;131(5):1541-9.

30.	Huang Y, Ouyang Y-Q, Redding SR. Previous breastfeeding experience and its influence on breastfeeding outcomes in subsequent births: a systematic review. Women and Birth. 2019;32(4):303-9.

31.	Steiner WA, Ryser L, Huber E, Uebelhart D, Aeschlimann A, Stucki G. Use of the ICF model as a clinical problem-solving tool in physical therapy and rehabilitation medicine. Physical therapy. 2002;82(11):1098-107.

32.	Arab-Zozani M, Hashemi F, Safari H, Yousefi M, Ameri H. Health-related quality of life and its associated factors in COVID-19 patients. Osong public health and research perspectives. 2020;11(5):296.

33.	Tomaso CC, Johnson AB, Nelson TDJS. The effect of sleep deprivation and restriction on mood, emotion, and emotion regulation: Three meta-analyses in one. Sleep. 2021;44(6):zsaa289.

34.	Abuhammad S AK, Khabour OF, Hamaideh S, Khasawneh B Mental Health of COVID-19 Recovered Individuals: A National Study from Jordan. electronic Journal of General Medicine. 2023;12.

35.	Carfì A, Bernabei R, Landi F. Persistent symptoms in patients after acute COVID-19. Jama. 2020;324(6):603-5.

36.	Méndez R, Balanzá‐Martínez V, Luperdi SC, Estrada I, Latorre A, González‐Jiménez P, et al. Short‐term neuropsychiatric outcomes and quality of life in COVID‐19 survivors. Journal of internal medicine. 2021;290(3):621-31.

37.	Mazza MG, De Lorenzo R, Conte C, Poletti S, Vai B, Bollettini I, et al. Anxiety and depression in COVID-19 survivors: Role of inflammatory and clinical predictors. Brain, behavior, and immunity. 2020;89:594-600.

38.	Xiong Q, Xu M, Li J, Liu Y, Zhang J, Xu Y, et al. Clinical sequelae of COVID-19 survivors in Wuhan, China: a single-centre longitudinal study. Clinical microbiology and infection. 2021;27(1):89-95.

39.	Hao F, Tam W, Hu X, Tan W, Jiang L, Jiang X, et al. A quantitative and qualitative study on the neuropsychiatric sequelae of acutely ill COVID-19 inpatients in isolation facilities. Translational psychiatry. 2020;10(1):355.

40.	Chen X, Chen H. Differences in preventive behaviors of COVID-19 between urban and rural residents: lessons learned from a cross-sectional study in China. International journal of environmental research and public health. 2020;17(12):4437.

41.	Wu C, Hu X, Song J, Yang D, Xu J, Cheng K, et al. Mental health status and related influencing factors of COVID‐19 survivors in Wuhan, China. Clinical and translational medicine. 2020;10(2):e52.
