# Supplementary material for: Physical and mental health well-being of COVID-19 recovered patients: A phenomenological study
Source: PLoS One. 2025 Aug 26;20(8):e0324433. doi: 10.1371/journal.pone.0324433 (PMC12380359; doi:10.1371/journal.pone.0324433)
Supplement: Attachment — Submitted filename: Response to Reviewers PLOS (1).docx [file pone.0324433.s002.docx]

**Response to Reviewers**

**Comments to the Author**

1. If the authors have adequately addressed your comments raised in a previous round of review and you feel that this manuscript is now acceptable for publication, you may indicate that here to bypass the “Comments to the Author” section, enter your conflict of interest statement in the “Confidential to Editor” section, and submit your "Accept" recommendation.

Reviewer #3: (No Response)

Reviewer #4: (No Response)

Reviewer #5: (No Response)

2. Is the manuscript technically sound, and do the data support the conclusions?

The manuscript must describe a technically sound piece of scientific research with data that supports the conclusions. Experiments must have been conducted rigorously, with appropriate controls, replication, and sample sizes. The conclusions must be drawn appropriately based on the data presented.

Reviewer #3: Partly

Reviewer #4: Partly

Reviewer #5: Yes

3. Has the statistical analysis been performed appropriately and rigorously?

Reviewer #3: N/A

Reviewer #4: Yes

Reviewer #5: Yes

4. Have the authors made all data underlying the findings in their manuscript fully available?

The [PLOS Data policy](http://track.editorialmanager.com/CL0/http:%2F%2Fwww.plosone.org%2Fstatic%2Fpolicies.action%23sharing/1/010f01951aa5025c-7590ab4e-f650-4d5a-acf6-e577dd79daa4-000000/mxUee6CBw94-z3fZe0FabvPFUjyuSwmBQgSwzgbiTV8=199) requires authors to make all data underlying the findings described in their manuscript fully available without restriction, with rare exception (please refer to the Data Availability Statement in the manuscript PDF file). The data should be provided as part of the manuscript or its supporting information, or deposited to a public repository. For example, in addition to summary statistics, the data points behind means, medians and variance measures should be available. If there are restrictions on publicly sharing data—e.g. participant privacy or use of data from a third party—those must be specified.

Reviewer #3: Yes

Reviewer #4: Yes

Reviewer #5: Yes

5. Is the manuscript presented in an intelligible fashion and written in standard English?

PLOS ONE does not copyedit accepted manuscripts, so the language in submitted articles must be clear, correct, and unambiguous. Any typographical or grammatical errors should be corrected at revision, so please note any specific errors here.

Reviewer #3: Yes

Reviewer #4: No

Reviewer #5: No

6. Review Comments to the Author

Please use the space provided to explain your answers to the questions above. You may also include additional comments for the author, including concerns about dual publication, research ethics, or publication ethics. (Please upload your review as an attachment if it exceeds 20,000 characters)

Reviewer #3: Dear authors
Thank you for your efforts to do this research. Below are some comments and suggestions to help improve the clarity and impact of your study.
The authors discuss the COVID-19 Survivor Parents' Physical and Mental health well-being; however, there is no clear explanation as to why parents specifically were chosen as the focus of the study. Furthermore, simply considering parents as a group is insufficient. The term "parents" is broad and encompasses various subgroups. It is not clear what criteria were used to define parents in the study. For instance, the criteria of being hospitalized or receiving oxygen therapy are not distinguishing enough. The study could benefit from distinguishing among different types of parents, such as those with healthy children, those with children who have chronic conditions, those who lost a child to COVID-19, and those with children who require transplants. Each of these subgroups would likely have different experiences.

The study appears to treat all COVID-19 survivor parents as a homogeneous group, as if investigating the experiences of ordinary survivors without considering the diversity among them. Additionally, in the introduction, the authors state, "Many survivor parents of COVID-19 suffer from lingering physical symptoms, often referred to post-COVID-19(14, 15)". However, the references reviewed did not specifically address parents.

Reviewer #4: The paper presents the findings of an intriguing qualitative study on the experience of patients who have recovered from Coronavirus Disease 2019 with respect to their physical and psychological quality of life. The topic is both timely and of significant interest; however, it is recommended that responses address the methodology and the use of excessive diagnostic terminology, and that the paper be proofread by native speakers to enhance its scientific English.

Reviewer #5: Note: I reviewed the first manuscript in the submission. There was a “revised” manuscript at the end that was not reviewed or compared to the first.

This qualitative study examined the occurrence of persistent post-COVID-19 health issues using unstructured interviews in patients who had severe COVID-19.

The study was sufficiently methodically sound to ensure the results have low bias and are valid across raters.

The discussion/conclusion could be improved; please explicitly tell what you found, what you expected, any differences between the two and point out any new unexpected themes. These aspects are somewhat present, but please work on making them more explicit.

There are a large number of small grammatical and language issues that I suspect are a result of the authors not having English as their first language. These should be fixed in order to make the manuscript appear professional. There are so many, I stopped trying to note each one. I recommend hiring or otherwise utilizing a writer experienced scientific writing in English to do a full pass to fix these issues. Here is the incomplete list of items I identified before I stopped, use the search function to locate the problem areas I identified:

• Introduction of abstract. Break into 2 or more sentences. Opening sentence is not clear, I think you mean “Understanding Post-severe infection health…”. Revise.
• In the abstract results, the sentence, “The cognitive health subheading include subheadings such as loss of memory and diminish ability of focusing and changes in cognitive abilities.” should only mention subheadings once. Revise
• Do not use colloquial language. Here are some phrases that need to be replaced with precise scientific language.
o “sobering light” – being sober or not is irrelevant.
o “treacherous path”. --- they are not literally walking on a path.
o “grapples” / “grappling --- I don’t think wrestling is involved
o “illuminating”
o “forge a path”
o “Embark on a journey”
• Replace “This subject studying” with “This study”. Pg. 10
• In, “Many survivor parents of COVID-19 suffer from
• lingering physical symptoms, often referred to post-COVID-19(14, 15).” The last part of this sentence is not clear.
• “The impact of COVID-19 pandemic is profound…”. Should be “The impact of THE COVID-19”
• “and all survivors that includes parents showed suffering from mental health issues”. Remove “that includes parents” I’m not sure why this is in there.
