# Supplementary material for: Physical and mental health well-being of COVID-19 recovered patients: A phenomenological study
Source: PLoS One. 2025 Aug 26;20(8):e0324433. doi: 10.1371/journal.pone.0324433 (PMC12380359; doi:10.1371/journal.pone.0324433)
Supplement: Attachment — Submitted filename: Response to Reviewer PLOS ONE.docx [file pone.0324433.s003.docx]

Response to Reviewers

Reviewer #4: The authors have correctly revised the paper in accordance with the reviewer's instructions. The text has undergone a thorough proofreading process, resulting in enhanced fluency.

Thank you so much

Reviewer #5: The manuscript language is very much improved. Thanks for your excellent contribution and congratulations on publishing!

Thank you so much

Reviewer #6: The study addresses an important topic. The aim of the study is clearly stated, and the focus on both physical and mental health aspects is crucial for understanding the holistic recovery of individuals post-infection.

The use of a qualitative approach, particularly unstructured interviews, is appropriate for exploring participants' experiences in-depth. This method allows for a nuanced understanding of the subjective experiences of individuals recovering from COVID-19, which is critical for informing both clinical practice and future research.
Thank you so much

However, I have a few suggestions and questions regarding the study's methodology:

Subject Selection and Sampling:
Could the authors specify how many hospitals were selected for participant recruitment and how many patients were selected from each hospital? This detail will help readers understand the scope and diversity of the sample.

Added

Sampling Method and Sample Size:
It would be beneficial if the authors could describe the sampling method in more detail. Additionally, it would be helpful to explain why a sample size of 30 participants was chosen.

Added

Use of Qualitative Software:
If a qualitative software was used to analyze the data, the authors should provide more details on how it was utilized (e.g., for coding or data organization). Additionally, citing the software's references and giving a brief explanation of its role in the analysis would enhance the transparency of the research process.

Added

Participant Compensation:
Were participants compensated for their participation in the study? If so, it would be helpful to mention this information to ensure transparency and address ethical considerations. If not, an explanation of the rationale for not compensating participants would be appreciated.

Added

Conclusion:
The conclusion would benefit from a more detailed discussion of specific recommendations

Added
